# Impaired TFEB-mediated autophagy-lysosome fusion promotes tubular cell cycle G2/M arrest and renal fibrosis by suppressing ATP6V0C expression and interacting with SNAREs

**DOI:** 10.7150/ijbs.91480

**Published:** 2024-03-03

**Authors:** Xiang Ren, Jing Wang, Huizhi Wei, Xing Li, Yiqun Tian, Zhixian Wang, Yisheng Yin, Zihao Guo, Zhenliang Qin, Minglong Wu, Xiaoyong Zeng

**Affiliations:** 1 Department of Urology, Tongji Hospital, Tongji Medical College, Huazhong University of Science and Technology, Wuhan, China.; 2 Hubei Provincial Institute of Urology, Wuhan, China.; 3 School of Pharmaceutical Science, Shanxi Medical University, Taiyuan, China.; 4 Shanxi Key Laboratory of Innovative Drug for the Treatment of Serious Diseases Basing on the Chronic Inflammation, Taiyuan, China.; 5 Department of Urology, Wuhan Hospital of Traditional Chinese and Western Medicine, Tongji Medical College, Huazhong University of Science and Technology, Wuhan, China.; 6 Department of Orthopaedics, Tongji Hospital, Tongji Medical College, Huazhong University of Science and Technology, Wuhan, China.

**Keywords:** Renal fibrosis, transcription factor EB, autophagy, cell cycle, V-ATPase, methylation

## Abstract

Increasing evidence suggests that autophagy plays a major role during renal fibrosis. Transcription factor EB (TFEB) is a critical regulator of autophagy- and lysosome-related gene transcription. However, the pathophysiological roles of TFEB in renal fibrosis and fine-tuned mechanisms by which TFEB regulates fibrosis remain largely unknown. Here, we found that TFEB was downregulated in unilateral ureteral obstruction (UUO)-induced human and mouse fibrotic kidneys, and kidney-specific TFEB overexpression using recombinant AAV serotype 9 (rAAV9)-TFEB in UUO mice alleviated renal fibrosis pathogenesis. Mechanically, we found that TFEB's prevention of extracellular matrix (ECM) deposition depended on autophagic flux integrity and its subsequent blockade of G2/M arrest in tubular cells, rather than the autophagosome synthesis. In addition, we together RNA-seq with CUT&Tag analysis to determine the TFEB targeted gene ATP6V0C, and revealed that TFEB was directly bound to the ATP6V0C promoter only at specific site to promote its expression through CUT&Run-qPCR and luciferase reporter assay. Interestingly, TFEB induced autophagic flux integrity, mainly dependent on scaffold protein ATP6V0C-mediated autophagosome-lysosome fusion by bridging with STX17 and VAMP8 (major SNARE complex) by co-immunoprecipitation analysis, rather than its mediated lysosomal acidification and degradation function. Moreover, we further investigated the underlying mechanism behind the low expression of TEFB in UUO-induced renal fibrosis, and clearly revealed that TFEB suppression in fibrotic kidney was due to DNMT3a-associated TFEB promoter hypermethylation by utilizing methylation specific PCR (MSP) and bisulfite-sequencing PCR (BSP), which could be effectively recovered by 5-Aza-2'-deoxycytidine (5A-za) to alleviate renal fibrosis pathogenesis. These findings reveal for the first time that impaired TFEB-mediated autophagosome-lysosome fusion disorder, tubular cell G2/M arrest and renal fibrosis appear to be sequentially linked in UUO-induced renal fibrosis and suggest that DNMT3a/TFEB/ATP6V0C may serve as potential therapeutic targets to prevent renal fibrosis.

## Introduction

Chronic kidney disease (CKD) is a progressive renal dysfunction that can be caused by prolonged ureteral obstruction and is becoming one of the common and costly worldwide public health problems 1, 2. Renal fibrosis with the characteristic of excessive extracellular matrix (ECM) deposition, is a central terminal manifestation and irreversible pathological process by which CKD progresses into end-stage renal disease (ESRD) 3, in which dysregulated repair of tubular epithelium is a significant pathological mechanism of renal dysfunction 4-6 and the proximal tubular cell is considered to be a key initiating factor 7-9. Moreover, epithelial-mesenchymal transition (EMT) stands as the most important cause and primary driver of renal fibrosis and human renal proximal tubular cell line HK-2 is desired and believed as a widely-used model to clarify the underlying mechanisms of tubular epithelium EMT and renal fibrosis in previous studies 10-13. Importantly, despite the growing comprehension of the pathogenesis of renal fibrosis, there remains a lack of effective therapies capable of arresting the progression of CKD to ESRD.

Autophagy, an evolutionarily conserved process facilitated by lysosomes to degrade damaged subcellular organelles and cellular proteins 14, is widely recognized as one of the main responders in the pathogenesis of kidney injury 12,13. In response to stress, cells can protect themselves and maintain tissue homeostasis by engaging cellular protective mechanisms, such as activating lysosomal biogenesis and autophagy 15. Numerous studies have confirmed that autophagy is stimulated in proximal tubular cells and serves as an innate protective mechanism in acute and chronic kidney diseases 16-18, and dysregulation of autophagy is confirmed to be the main cause of cellular damage 19, 20. The transcription factor EB (TFEB), a pivotal controller of lysosome biogenesis and autophagy-related genes 21, can coordinate an effective transcriptional program through dephosphorylation, activation and nuclear translocation to upregulate genes responsible for both the early and late stages of autophagy, in line with the heightened requirement for autophagic degradation 22. It has been demonstrated that the expression of TFEB was lowered in human idiopathic pulmonary fibrosis tissues and chronic ethanol-induced liver injury 23, 24, and pharmacological activation or overexpression of TFEB improved autophagic flux and developed less severe pulmonary fibrosis and liver injury. Moreover, the positive anti-fibrotic effect of TFEB-mediated autophagy also be observed in recent progress in different model systems 22, 25. Thus, these findings indicate the vital involvement of the TFEB-autophagy signaling in the development of renal tubular injuries and fibrosis pathogenesis. Based on the tight association between autophagy and fibrogenesis, the causal role of TFEB in ureteral obstruction-induced kidney fibrosis and fine-tuned mechanisms by which TFEB regulates fibrosis should be deciphered.

Lysosomes performing a crucial role in the process of autophagy, including lysosomal acid degradation and autophagosome-lysosome fusion, and previous studies have revealed that TFEB-mediated regulation of lysosomal function acts as a protective mechanism against autophagy dysregulation 16, 26. Notably, the lysosome is a critical and terminal component of autophagy, housing more than 50 acid hydrolases 21, 27. Imbalances in acidification and increased intraluminal pH can impede enzyme activity within the lysosome, hindering the removal of substrates such as protein aggregates and damaged organelles 28. Importantly, the lysosome not only serves as a site for acid degradation during autophagy but also supplies sectional SNARE proteins that participate in membrane fusion with autophagosomes, constituting the final step of autophagy 29. The vacuolar-type HC-ATPase (V-ATPase) complex, a well-known proton pump that uses a rotary mechanism through the V1 and V0 domains, is responsible for creating and maintaining the lysosomal acidic environment 30. ATP6V0C, a key constituent of the V0 domain of V-ATPase, is responsible for constructing the proteolipid c-ring of the V-ATPase. It plays a critical role in generating proton gradients within synaptic vesicles and regulating intra and extracellular pH value 31, 32. However, whether and how between ATP6V0C and the SNARE synaptobrevin interact is largely unknown. Moreover, there is no available data regarding the direct regulatory role of TFEB on ATP6V0C expression and its specific mechanisms in the context of renal fibrosis.

Here, we found that TFEB protein was decreased in both human and mouse of unilateral ureteral obstruction (UUO)-induced fibrotic kidney, and that overexpression of TFEB in mouse kidneys attenuated fibrogenesis. Moreover, we found that TFEB prevented ECM deposition mainly through blocking tubular cell G2/M arrest, which was tightly dependent on autophagic flux integrity but not autophagosome synthesis. Interestingly, we uncovered that TFEB was directly bound to the ATP6V0C promoter at Site C by combing RNA-seq and CUT&Tag analysis and TFEB induced autophagic flux integrity, mainly dependent on scaffold protein ATP6V0C-mediated autophagosome-lysosome fusion by bridging with STX17 and VAMP8 (major SNAREs), rather than its mediated lysosomal acidification and degradation function. Lastly and impressively, we further investigated the underlying mechanism behind the low expression of TEFB in UUO-induced renal fibrosis, and clearly revealed that TFEB suppression was due to DNMT3a-associated TFEB promoter hypermethylation, which could be effectively recovered by 5-Aza-2'-deoxycytidine (5A-za) to alleviate renal fibrosis pathogenesis.

## Materials and Methods

### Animal studies

We obtained male C57BL/6 mice (20-25g) aged 6-8 weeks from the Hubei Provincial Centers for Disease Control and Prevention in Wuhan, China. To establish the unilateral ureteral obstructed (UUO) models, we performed unilateral ureteral ligation following established protocols. The C57BL/6 mice were then randomly assigned to sham surgery group, UUO-3d group, UUO-7d group, and UUO-14d group. For the 5A-za (Sigma-Aldrich, USA) intervention, both sham mice and UUO mice were intraperitoneally injected (0.35 mg/kg, kilogram bodyweight) at one day prior to surgery and then daily injection for 14 days. The animals were sacrificed at different intervals and the blood and renal samples were collected for various analyses. All animal studies were performed in accordance with the approved protocol of the Animal Care and Use Committee of Tongji Medical College, Huazhong University of Science and Technology and approved by the Ethics Committee of Tongji Medical College Animal Care Center (Number: No. TJ-C20210145).

### Intrarenal adeno-associated virus (AAV) delivery

Overexpression of adeno-associated virus (AAV) constructs of TFEB (rAAV9-TFEB) with a vector of AAV-NPHS1-MCS-T2A-LUC was designed and purchased from GENERAL BioL (Anhui, China). The titer of rAAV9-TFEB was approximately 1.0 × 10^13^ viral genomes/mL, and were delivered to the kidney through tail vein injection 100 μl per mouse. Preliminary studies demonstrated that, after one month, AAV-mediated protein expression in the kidney parenchyma significantly increased. And infection efficiency in the kidney was verified by immunofluorescence and Western blotting analysis. After those, the sham surgery or UUO model was established.

### Cell culture and treatment

Human proximal tubule epithelial cell line (HK-2) cells were cultured in a humidified environment at 37 °C with 5% CO_2_. The culture medium consisted of DMEM/F12 (Boster, China) supplemented with 10% fetal bovine serum (FBS, Gibico, USA). HK-2 cells were treated with 10 ng/ml TGF-β1 (Sigma-Aldrich, USA) for 48h to induce fibrotic changes. Before some other treatments, cells were starved for 24 hours using a medium containing 0.5% FBS. For the stimulation of cell cycle, the cells were treated with 0.5 mmol/L hydroxyurea (MCE, USA) or 0.25 ug/ml nocodazole (MCE, USA) for 20 h. For the detection of autophagy, the cells were pretreated with 10 mg/ml E64d-pepstatin A 60 min, 10 nM Rapamycin (MCE, USA) 24h or 150 nM Bafilomycin A1 12h, and then stimulated with TGF-β1 for 48h. To induce demethylation, the cells were pre-treated with 5A-za (10 mmol/L) for 24 h, followed by incubation with TGF-β1 for 48h. For the culture of HEK-293T cells, they were resuspended and cultured in DMEM (Boster, China) medium supplemented with 10% fetal bovine serum and antibiotics at 37 °C and 5% CO_2_.

### Human renal tissues

Renal tissues were obtained from renal cortex of six patients receiving nephrectomy or partial nephrectomy surgery. Fibrosis renal tissues were from three patients diagnosed with CKD and nonfunctional kidney because of ureteral stone obstruction. The normal control renal samples were from the sex and age-matched three patients suffered from with renal clear cell carcinoma (T1aN0M0). The diagnosis was in accordance with the AJCC criteria for renal cancer 2017. Specially, the tissue samples were acquired from at least 1cm away from the margin of the tumor. Informed consent from all patients was obtained and all experimental protocols were approved by Tongji Hospital licensing committee (Number: No. TJ-IRB20210205).

### Cell transfection

Lentiviruses for TFEB overexpression (LV-TFEB, NM_001167827.3) and ATP6V0C silencing (LV-shATP6V0C, NM_001694) Along with their corresponding negative controls were designed and constructed by GENERAL BioL (Anhui, China). HK-2 cells were transfected with specific lentiviruses (including the lentivirus carrying the mRFP-GFP-LC3 plasmid) at a multiplicity of infection (MOI) of 20 in the presence of polybrene (Genomeditech, China) for 24 h. The cells were then cultured in complete medium for two days and treated with 2.5 μg/ml of puromycin (Sigma-Aldrich, USA) to establish stable cell lines. For siRNA transfection, Lipofectamine 3000 (Invitrogen, USA) was used to transfect siTFEB or siATP6V0C (GENERAL, China) at a concentration of 100 nmol/L, or a scrambled control siRNA, into HK-2 cells in OptiMEM (Invitrogen, USA).

### Histology, immunohistochemical and immunofluorescence staining

The renal tissues were excised, fixed in 4% buffered formalin, embedded in paraffin, and sectioned (4 mm) by a routine procedure. Hematoxylin and eosin (H&E) staining, Masson's trichrome staining, immunohistochemical staining and immunofluorescence staining were conducted according to routine protocols and observed by light microscopy (Leica DMI6000B, Germany). For quantitation of immunofluorescence colocalization, the images were taken from ImageXpress Micro four High Content Imaging System with Ph1 S Plan Fluor ELWD ADM 20XC objective and Andor SDK3 camera (Molecular Devices, USA) and analyzed using MetaXpress analysis software.

### Western blot analysis

RIPA buffer (Servicebio, China) was used to extract total protein from tissues or cells, and the protein concentration was determined using a bicinchoninic acid assay kit (Boster, China). Protein samples were subjected to electrophoresis and transferred onto a polyvinylidene fluoride membrane. The protein blots were then incubated with the specified primary antibody overnight at 4 °C, as following: anti-α-SMA, anti-Collagen I, anti-N-cadherin, anti-Fibronectin, anti-E-cadherin, anti-Vimentin, anti-β-actin, anti-LC3, anti-p62 and anti-GAPDH (all from Abclonal, China), anti-TFEB, anti-ATP6V0C, anti-CDKN1A, anti-CCNB1, anti-STX17, anti-SNAP29, anti-VAMP8, anti-DNMT3a and anti-DNMT3b (all from Proteintech, USA), anti-p-CDK1 and anti-DNMT1 (both from CST, USA) antibodies, and subsequently incubated with the appropriate secondary antibody horseradish peroxidase (HRP)-conjugated anti-mouse or anti-rabbit immunoglobulin G (IgG) (SAB, USA) for 1h. Blots were visualized through the enhanced chemiluminescence detection system (Bio-Rad, USA).

### Quantitative real-time PCR and RNA sequencing (RNA-seq)

Total RNA from HK-2 cells was extracted using the TRIzol reagent (Invitrogen, USA) and the concentration was determined by a Nanodrop2000 (Thermo Fisher Scientific, USA). cDNA was synthesized using a Reverse Transcription Kit (Vazyme, China) following the manufacturer's instructions. Quantitative real-time PCR (qRT-PCR) reactions were performed using a SYBR Green Master Mix (Vazyme, China) by a QuantStudio 5 instrument (Biosystems, USA). The relative expression was calculated with the equation 2^-ΔΔCT^. The primer sequences used were as following: TFEB-F: CAGCAGTCGCAGCATCAGAAGG, TFEB-R: TGTTGCCAGCGGAGGAGGAC; ATP6V0C-F: CAACGCTGCGGAGATCCA, ATP6V0C-R: CAGCTGGAGGAAGCTCTTGT.

For RNA-seq, total RNA detection, gene library construction, and high-throughput sequencing were conducted following the manufacturer's guidelines (Major Biotechnology, China) on the Illumina NovaSeq 6000 platform.

### Methylation specific PCR (MSP) and bisulfite-sequencing PCR (BSP)

Genomic DNAs were extracted and purified using DNA extraction and purification Kit (Vazyme, China), and the purified DNA sample was bisulfite-modified using EpiArt DNA Methylation Bisulfite Kit (Vazyme, China) following the instructions. For mouse TFEB promoter, we used methylated primer mMetF: GTGATAGTCGGAGTTTAAGCGTC; mMetR: ACAACTTATCCCAAATTCGAA and unmethylated primer mUmetF: GGAGTGATAGTTGGAGTTTAAGTGTT; mUmetR: CAACTTATCCCAAATTCAAA. For internal control (Input), the forward primer was mInF: GGACGCAGAGAACGGAGAC and reverse primer was mInR: CCAGGTAGGACTGCACCTTC. MSP for human HK-2 cells and renal tissues, we used methylated primer hMetF: GTGGAGTGATAGTCGGAGTTC; hMetR: GACAACTTATCGCAAATTCGAA and unmethylated primer hUmetF: GTGGAGTGATAGTTGGAGTTTGG; hUmetR: ACTCCAACAACTTATCACAAATTCA. The Input primers were hInF: GTCCAGCAACATGACAGCAA and hInR: GGTAGGACTGCACCTTCAACA. The PCR products were analyzed on a 1.5% agarose gel and the band densitometry was quantified ImageJ software.

For BSP, bisulfite-treated genomic DNA from mouse renal tissues were was amplified using BSP forward primer: ACTGGCCGTCGTTTTAC and reverse primer: CAGGAAACAGCTATGAC, and the amplified PCR products were gel-purified and cloned into pCE2 TA/Blunt-Zero Vector (Vazyme, China). Five single colonies for each PCR were randomly selected for sequencing (Qingke, China). The percentages of methylated cytosines to total cytosines in the cloned fragment were then calculated.

### Chromatin immunoprecipitation sequencing (ChIP‑seq) and CUT&Tag

The ChIP‑seq assay was conducted with a CUT&Tag Assay Kit (Vazyme, China) following the manufacturer's instructions. Briefly, 1×10^6^ HK-2 cells were collected and their nuclei were isolated. The isolated nuclei were fixed onto activated conA beads and incubated with anti-TFEB antibodies in DIG buffer at 4 °C overnight. After washing, the bound antibodies were incubated with secondary antibody for 1 h at 25 °C and then treated with 0.04 μM Hyperactive pA/G-Tnp adapter complex at 25 °C for 1 h. The interactions were quenched by adding Proteinase K, Buffer L/B, and DNA extraction beads and were incubated at 55 °C for 10 min. Beads were gently washed with ethanol and DNA was resuspended with double-distilled water. The TruePrep Index Kit V2 for Illumina (Vazyme, China) was used for DNA library amplification. Then, the DNA fragments were amplified using PCR with the procedure with 72 °C for 3 min, followed by 10 cycles of denaturation at 98 °C for 10 seconds, annealing at 60 °C for 5 s, and extension at 72 °C for 1 min. The final step was holding at 4 °C. The VAHTS DNA Clean Beads (Vazyme, China) were added and purified the PCR products and extracted library. The last CUT&Tag libraries were sequenced by HaploX (Jiangxi, China) using the Illumina PE150 platform.

### Chromatin immunoprecipitation qPCR (ChIP‑qPCR) and CUT&Run-qPCR

The ChIP‑qPCR assay was conducted with a CUT&Run Assay Kit following the procedure (Vazyme, China) according to the manufacturer's instructions. Similarly with CUT&Tag assay, 1×10^6^ HK-2 cells were collected, and their nuclei were isolated and purified with activated conA beads and incubated with an anti-TFEB antibody or a rabbit IgG (internal control control) overnight at 4 °C. Nuclei were incubated with pG-MNase Enzyme for 1 h at 4 °C the next day. Then CaCl_2_ was added and incubated for 90 minutes at 0 °C to promote MNase digestion. The fragments were stopped by adding Stop Buffer and were washed with ethanol, and DNA was resuspended with double-distilled water. Subsequently, qPCR was performed to detect target genes. Primers for CUT&Run-qPCR were as follows: ATP6V0C-A-F: GGAAGAATGAGGGCGTCTGA, ATP6V0C-A-R: CAAGTGGACCTCTGTGCTGC; ATP6V0C-B-F: CTTCTCAGAAACAAGGGCCG, ATP6V0C-B-R: GGTTCAAAGGAGAGGAGGCAA; ATP6V0C-C-F: CTGAAGGTGCAGGCTTTGC, ATP6V0C-C-R: TGTCTCTGCCCCTCAAGATG and ATP6V0C-D-F: AGGCAGTAGGTCTGGGTCTG; ATP6V0C-D-R: CACAGCGTCCGGAAAAGC.

### Luciferase reporter assay

The luciferase pPRO-RB-Report vector containing the complete promoter sequence of ATP6V0C (Promoter- ATP6V0C), the TFEB expression plasmid (TFs-TFEB) and the negative control vector (Promoter-NC and TFs-NC) were constructed by GENERAL BioL. The vectors carrying four mutated points were also constructed by GENERAL BioL. HEK293T cells were co-transfected with luciferase reporter vector, TFs-TFEB or controls using Lipofectamine 3000 (Invitrogen, USA). After transfection for 48h, luciferase activity was measured using the Dual-Luciferase Reporter Assay System (Promega, USA).

### Co-immunoprecipitation (co-IP)

The co-IP assay was performed following the manufacturer's instructions (Abbkine, China). Briefly, 5×10^6^ HK-2 cells were lysed in Non-Denaturing Lysis Buffer, and the cell lysates were incubated with relative primary antibodies or normal rabbit IgG and then mixed withProtein A/G Magnetic Beads for overnight at 4 °C. After washing the beads, the captured immune complexes were eluted with SDS-PAGE loading buffer. The results of the co-IP were detected by western blot assay using the corresponding primary antibodies.

### Lyso-tracker Red, Lyso-Sensor Green and DQ-OVA staining

Cells were seeded in 96-well plates, Lyso-tracker Red dye, Lyso-Sensor Green dye or FITC-DQ-OVA was added to each well. After incubation at 37 ºC for 1 h, the cells were washed 3 times with PBS, and for Lyso-tracker Red staining, the fluorescent images were taken using ImageXpress Micro four High Content Imaging System with Ph1 S Plan Fluor ELWD ADM 20XC objective and Andor SDK3 camera (Molecular Devices, USA). For Lyso-Sensor Green and DQ-OVA staining, the fluorescence intensity was determined using flow cytometry (BD FACSAria, USA).

### Flow cytometry

The cells in various phases of the cell cycle were analyzed using flow cytometry with the Annexin cell cycle staining kits (Beyotime, China). Briefly, 1×10^6^ cells (per sample) were stained with Ribonuclease A and PI solution for 4 h in darkness. After washing, the percentages of stained cells were sorted using flow cytometry on the BD FACScan System (NJ, USA), and cell cycle analysis was performed using Flow Jo software (version 10.7.2).

### Transmission electron microscopy (TEM) and confocal microscopy

For TEM detection, mouse kidney tissues were fixed with 3% osmium tetroxide (OsO4) for 2 h, followed by embedding in Epon resin and sectioning into 100 nm pieces. The samples were then visualized using a TEM at 80 kV (Hitachi, Japan). The immunofluorescence colocalization for LC3 (1:250, Abcam) and LAMP1 (1:100, Abcam) were performed following the routine protocols and observed by using a laser scanning confocal microscope (Olympus, Japan), and the colocalization analyses were done using the Image J software. For the observation of cells transfected with specific lentiviruses mRFP-GFP-LC3, the samples were also imaged with a laser scanning confocal microscope (Olympus, Japan).

### Serum creatinine measurement

The creatinine was tested using test kits (Jiancheng, China) according to the manufacturer's instructions.

### Statistical analysis

All the data are represented as the mean ± standard deviation (SD) from three independent experiments. The differences between groups were determined using unpaired Student's *t*-test for two groups of data and one-way or two-way ANOVA with Tukey's multiple comparisons test or Dunnett's multiple comparisons for multiple groups of data. A *P* value of < 0.05 was considered statistically significant. All analysis was performed using GraphPad Prism version 9.0.0.

## Results

### TFEB is a potential anti-fibrosis factor and suppressed in fibrotic kidneys

**Figure 1:**
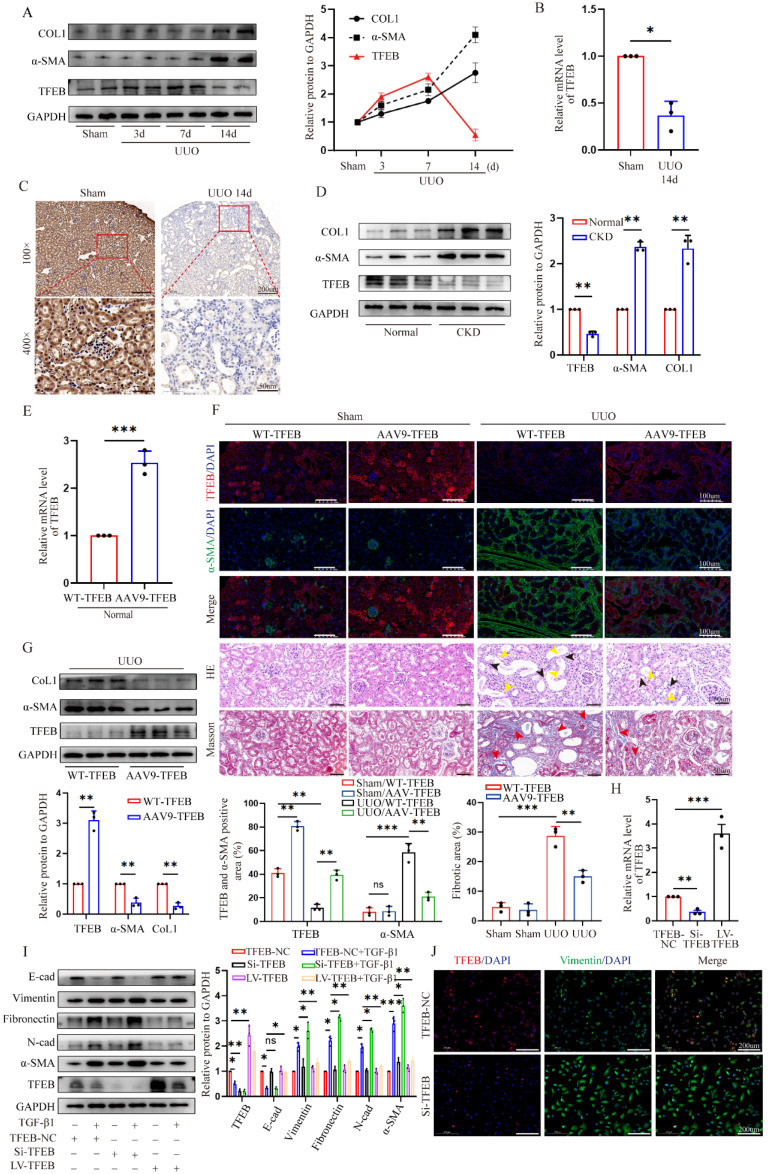
**TFEB is a potential anti-fibrosis factor and suppressed in fibrotic kidneys.** (A) Immunoblot and quantitative analysis of TFEB, α-SMA and CoL1 in renal tissues from mice on days 3, 7, and 14 after UUO and sham control surgery (Sham). Two randomly selected samples from each group were shown. (B) RT-qPCR analysis of mRNA levels of TFEB in renal tissues from sham and UUO mice after 14 days (UUO group). (C) Representative IHC for TFEB content in renal tissues. Scale bar, 200 μm and 50 μm. (D) Immunoblot and quantitative analysis of TFEB, α-SMA and CoL1 from renal tissues of CKD patients and normal controls. (E) The mRNA levels of TFEB in renal tissues isolated from wild type (WT-) and AAV9-TFEB treated control mice. (F) Representative double immunofluorescence staining and quantitative analysis of TFEB and α-SMA content in renal tissue from WT and AAV9-TFEB mice after UUO or sham surgery. Scale bar, 100 μm. H&E and Masson trichrome staining of renal tissues and quantifications of fibrotic areas. Scale bar, 50 μm. The black arrows indicate parenchymal loss, the yellow arrows indicate tubular atrophy, and the red arrows indicate collagen deposition and interstitial fibrosis. (G) Immunoblot and quantitative analysis of TFEB, α-SMA and CoL1 from renal tissues of WT- and AAV9-TFEB treated UUO mice after14 days. Three randomly selected samples from each group were shown. (H) RT-qPCR analysis of TFEB knockdown (Si-TFEB) or overexpression (LV-TFEB) efficacy compared to empty vector in the HK-2 cells. (I) Immunoblot and quantitative analysis of TFEB and EMT-related protein in TFEB control (empty vector, NC), downregulation or overexpression HK-2 cells incubated with or without TGF-β1. (J) Representative double immunofluorescence staining of TFEB and Vimentin in TFEB NC or downregulation HK-2 cells upon TGF-β1. Data are means ± SD at 3 independent experiments; ns, not significant, *p < 0.05, **p < 0.01, ***p < 0.001.

To gain insight into the pivotal role of TFEB in renal fibrosis pathogenesis, we first conducted a UUO mouse model. As expected, myofibroblast marker α-SMA and extracellular matrix protein collagen 1 (COL1) were markedly increased in fibrotic kidneys of UUO mice treated with for 14 days; Interestingly, TFEB expression levels were progressively elevated after under starvation of inflammatory phase but dramatically displayed a prominent repression at the peaked fibrosis formation at day 14 (Fig. 1A). Consistent with the protein levels, the mRNA levels of TFEB were decreased by the major development of fibrosis (Fig. 1B). Immunochemistry staining indicated that TFEB was enriched in almost all visible cell but significantly reduced in nuclei and whole tubular cell in fibrotic renal sections (Fig. 1C). Furthermore, we also investigated the expression levels of proteins from CKD patients. Consistently, Western blot demonstrated similar changes in protein levels (Fig. 1D). Thus, these results indicated that the expression repression of TFEB might be closely related to fibrosis development.

To investigate the effect of TFEB on renal fibrosis pathogenesis, recombinant AAV9-TFEB was efficiently transduced into the kidney through mice tail injection, resulting in kidney-specific over-expression of TFEB (Fig. 1E and F). The pathological staining of fibrotic kidneys showed that both wild type (WT-TFEB) and AAV9-TFEB UUO mice developed fibrotic lesions and displayed parenchymal loss, tubular atrophy, collagen deposition and interstitial fibrosis after obstruction for 14 days. Notably, AAV9-TFEB UUO mice downregulated α-SMA expression, and decreased tubular expansion and interstitial fibrosis in obstructed kidneys compared to UUO-matched TFEB WT mice (Fig. 1F). Accordingly, the immunoblot analysis also demonstrated that the expression levels of α-SMA and COL1 was dramatically reversed in AAV9-TFEB UUO mice (Fig. 1G).

Epithelial-to-mesenchymal transition (EMT) is widely recognized as a complete process of renal fibrosis. Hence, to look paralleling evidence for further validating the function of TFEB, we subsequently silenced and overexpressed TFEB with TFEB-siRNA or lentivirus plasmid for TFEB overexpression (LV-TFEB) in human renal epithelial cell line HK-2 *in vitro*. Real-time PCR and Western blot analysis indicated that the levels of TFEB expression were successfully inhibited and overexpressed, respectively (Fig. 1H and I). As anticipated, the Western blot analysis showed that the epithelial cell marker E-cadherin was notably downregulated and mesenchymal cell markers Vimentin, N-cadherin, Fibronectin and a-SMA were upregulated under treatment with transforming growth factor β1 (TGF-β1), an important mediator of tubulointerstitial fibrosis. More importantly, comparison with the control cells, TFEB knocking down partially exacerbated this change in EMT trend, while overexpression TFEB significantly alleviated the variation of EMT (Fig. 1I). Consistently, using the double immunofluorescence staining analysis, the results indicated that the knockdown of TFEB effectively enhanced the transition of Vimentin protein (Fig. 1J). Thus, these results reinforced that TFEB expression suppression was closely related to renal fibrosis development and it was protective against renal fibrosis.

### TFEB prevents ECM deposition through blocking tubular cell G2/M arrest

**Figure 2:**
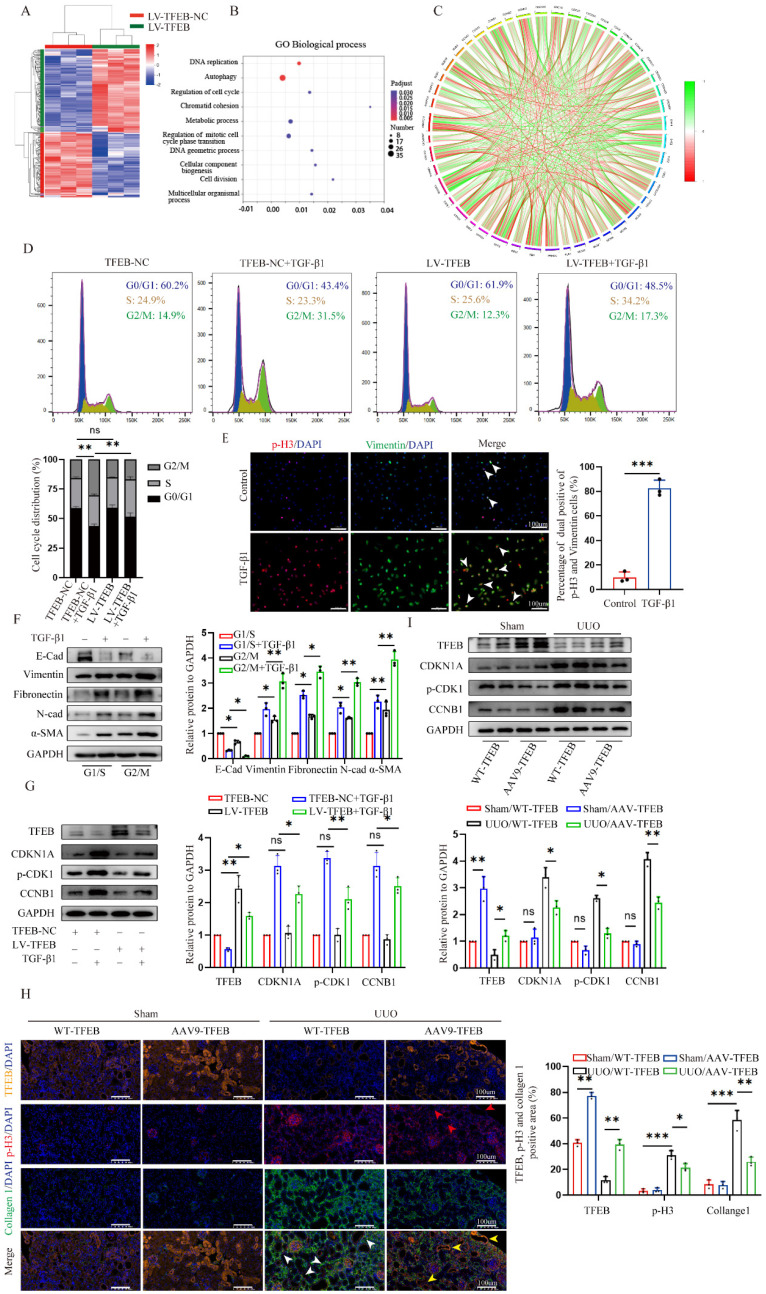
** TFEB prevents ECM deposition through blocking tubular cell G2/M arrest.** (A) Heatmap analysis of the top differentially expressed genes (DEGs) from RNA-seq analysis of TFEB NC and overexpression in HK-2 cells pretreated with TGF-β1. (B) Bubble plotted for the DEGs enriched in the different biological processes by GO analysis. (C) Different cell cycle‑related gene expression patterns in the TFEB-NC versus overexpression groups through Kyoto Encyclopedia of Genes and Genomes (KEGG) enrichment analysis. (D) Cell cycle distribution by flow cytometry analysis in TFEB-NC or overexpression HK-2 cells incubated with or without TGF-β1. (E) Representative double immunofluorescence staining and quantitative analysis of dual positive of p-H3 and Vimentin HK-2 cells treated with or without TGF-β1. Scale bar, 100 μm. The arrows indicate dual positive cells. (F) Cells synchronized at G1/S or G2/M phase by hydroxyurea or nocodazole were treated with or without TGF-β1, immunoblot and quantitative analysis of EMT relative protein. (G) Immunoblot and quantitative analysis of TFEB, CDKN1A, p-CDK1 and CCNB1 in TFEB-NC or overexpression HK-2 cells incubated with or without TGF-β1. (H) Representative treble immunofluorescence staining and quantitative analysis of TFEB, p-H3 and α-SMA content in renal tissues. Scale bar, 100 μm. The red arrows indicate TFEB^+^ and p-H3^-^ tubular cells. The white arrows indicate p-H3^+^ tubular cells with collagen accumulation. The yellow arrows indicate TFEB^+^ and p-H3^-^ tubular cells without collagen accumulation. Data are means ± SD, n = 3 independent experiments; ns, not significant, *p < 0.05, **p < 0.01, ***p < 0.001.

To investigate the underlying mechanism by which TFEB affects cell phenotypic switch to induce ECM, we conducted RNA-seq analysis of the control and TFEB overexpression HK-2 cells treated with TGF-β1. By applying a threshold (|log2 (fold change) | ≥ 0.7 and false discovery rate (FDR) < 0.05), there are a total of 3848 genes differentially expressed identified between the groups (Fig. 2A). Gene Ontology (GO) annotation highlighted biological processes involved in autophagy, DNA replication, and the cell cycle (Fig. 2B).

Furthermore, the differentially expressed genes enriched in the cell cycle pathway are well presented in Fig. 2C, suggesting that cell cycle might be crucial for the TFEB related ECM pathogenesis. Thus, to first evaluate which checkpoint tubular cell cycle distribution affected ECM accumulation, we performed flow cytometry to determine the distribution of cells in each phase and the results revealed that compared to the controls, TGF-β1 treatment caused more tubular cells arrested at G2/M phase (Fig. 2D). Furthermore, we analyzed mesenchymal development and the cell cycle arrest at the G2/M phase by co-staining Vimentin with phosphorylation of histone H3 at Ser10 (p-H3), and found the percentage of dual positive of p-H3 (G2/M phase arrest) and Vimentin was coordinately elevated after TGF-β1 treatment (Fig. 2E). Moreover, we further synchronized tubular cells at G1/S or G2/M phase by treatment with hydroxyurea or nocodazole. The Western blot analysis showed that the expression of Vimentin, N-cadherin, Fibronectin and a-SMA in G2/M-phase-synchronized cells was higher than that in G1/S-phase-synchronized cells in the presence or absence of TGF-β1 for 48 h, and E-cadherin behaved opposite results (Fig. 2F). Thus, these data virtually suggested that the enhanced G2/M arrest correlates with ECM accumulation in the tubular cells.

Next, we investigated the functional impact of TFEB on cell cycle progression. We found that TFEB overexpression obviously decreased the proportion of G2/M arrest cells upon TGF-β1 stimulation for 48 h (Fig. 2D). And, TFEB-overexpressing cells were accompanied by a significant reduction in the levels of CDKN1A/p21, phosphorylated (p)-CDK1 and CCNB1/cyclin B1 in the presence of TGF-β1 (Fig. 2G), indicating that TFEB negatively influenced on cell cycle progression at G2/M arrest. However, when HK-2 cells were in the absence of TGF-β1, neither control nor TFEB overexpression alone exhibited apparent effects on the distribution of the cell cycle or expression of cell cycle proteins (Fig. 2D and G), which confirmed again that TFEB-affected cell G2/M arrest was strongly associated with ECM pathogenesis. Next, we attempted to verify our findings through kidney-specific TFEB overexpression mice. As depicted in Figure 2I, very few tubular cells exhibited positive staining for p-H3 in both sham-operated WT- and AAV9-TFEB mice. However, after UUO 14 d, an increased number of p-H3-positive cells with elevated collagen were presented in WT-TFEB mice, while the response was effectively ameliorated in the AAV9-TFEB animals (Fig. 2H). Consistently, the levels of regulatory protein such as p21, p-CDK1 and cyclin B1 in UUO mice displayed similar pattern to that observed in tubular cells treated with TGF-β1 for 48 h. Remarkably, the production was reversed in AAV9-TFEB mice fibrotic kidney than that in WT-TFEB fibrotic kidney after UUO (Fig. 2I). Taken together, the deficiency of TFEB may contribute to kidney fibrosis through a mechanism involving G2/M arrest in the tubular cells.

### TFEB-mediated blocking of G2/M arrest depends on the autophagic flux integrity

**Figure 3:**
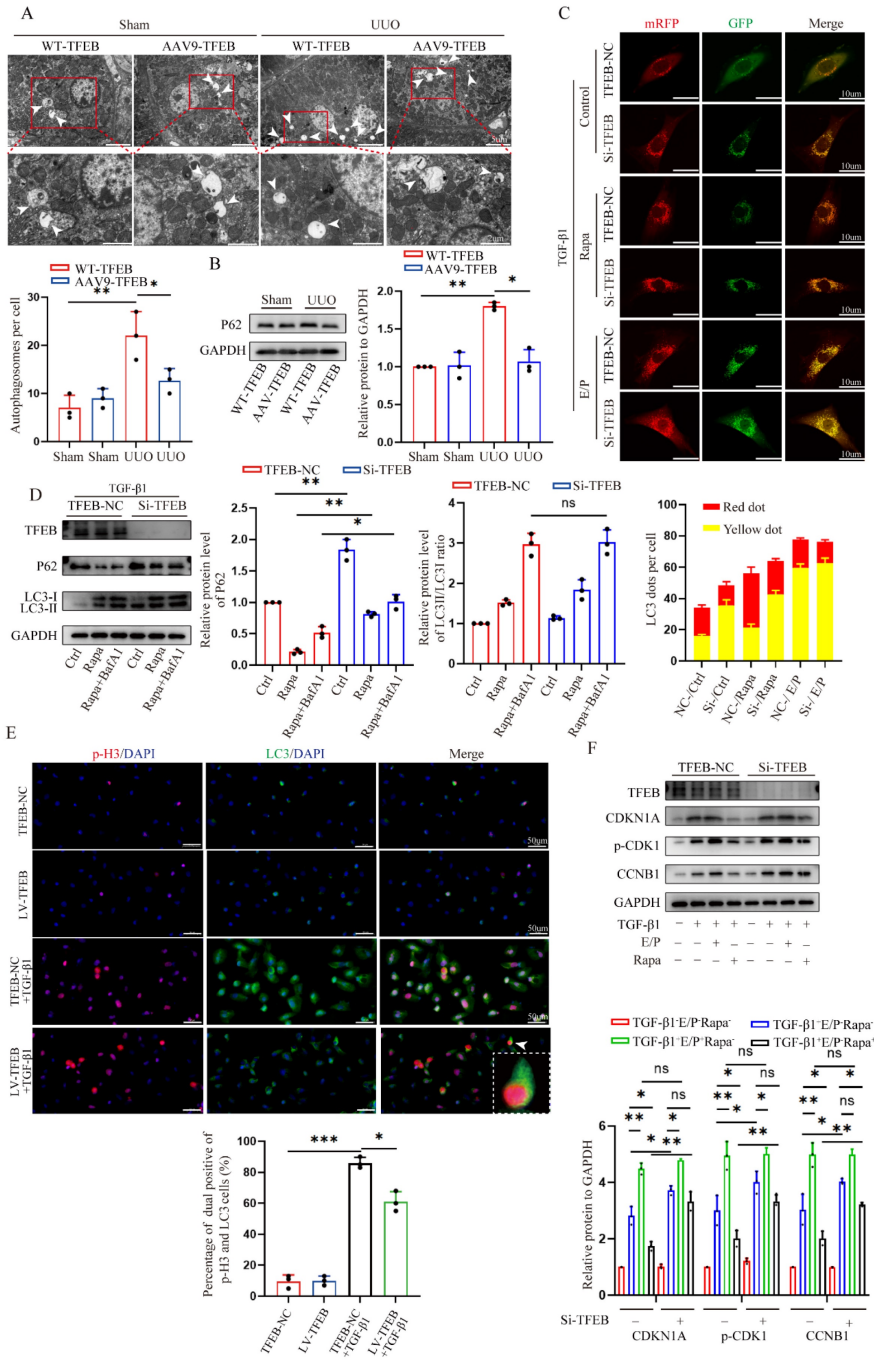
** TFEB-mediated blocking of G2/M arrest depends on integral autophagy flux.** (A) Representative images of autophagosome analysis by transmission electron microscopy in tubular cells of renal tissues. Scale bar, 5 μm and 2 μm. The arrows indicate autophagosome. (C) Representative images of HK-2 cells transfected with fluorescent mRFP‑GFP‑LC3. Scale bar, 10 μm. Quantitative analysis of red and yellow dots numbers. (D) Immunoblot and quantitative analysis of TFEB, P62 and ratio of LC3-Ⅱ/LC3-Ⅰ in TFEB-NC or knockdown HK-2 cells incubated with or without rapamycin or rapamycin and Bafilomycin A1. (E) Representative double immunofluorescence staining and quantitative analysis of dual positive of p-H3 and LC3 in TFEB-NC or knockdown HK-2 cells treated with or without TGF-β1. Scale bar, 50 μm. The arrow indicates dual positive cell. (F) Immunoblot and quantitative analysis of CDKN1A, p-CDK1 and CCNB1 in TFEB-NC or knockdown HK-2 cells incubated with or without TGF-β1, E/P or rapamycin. Data are means ± SD at 3 independent experiments; ns, not significant, *p < 0.05, **p < 0.01, ***p < 0.001.

In addition to being tightly associated with cell cycle regulation, TFEB has been playing a critical role in autophagy induction under starvation condition 21 (Fig. 2B). These previous findings encouraged us to investigate whether the induction of autophagy is necessary for TFEB-mediated cell cycle progression. We first directly assessed autophagosomes inside tubular cells in shamed and UUO WT- and AAV-TFEB mice using transmission electron microscopy (TEM). The results displayed that both shamed WT- and AAV9-TFEB mice had few autophagosome abundance (Fig. 3A), however, after UUO 14 d, markedly increased sealed double-membrane autophagosome accumulation was presented in TFEB WT animals, suggesting either an increased autophagosome synthesis or a defect in the completion of autophagy flux, whereas the accumulation was significantly decreased in the AAV9-TFEB mice (Fig. 3A). To this end, we continued to detected the expression levels of the SQSTM1/p62, which is a selective substrate of autophagy and could demonstrate the autophagy flux 33. As illustrated in Figure 3B, kidney tissue from wild- mice similarly exhibited a substantial elevation in the protein level of P62 after 14 days of UUO, while the response was significantly abrogated in the AAV-TFEB mice. To further clarify the effects of TFEB on autophagy integrity, we transduced a lentiviral plasmid encoding acid-resistant monomeric RFP and acid-sensitive GFP (mRFP-GFP-LC3B) into control and TFEB knockdown HK-2 cells (Fig. 3C).

More intuitively, under treatment with TGF-β1, more yellow (colocalized GFP^+^-RFP^+^) puncta were accumulated in the TFEB knockdown cells compared with that of control cells; after the induction of autophagy with rapamycin, the green puncta (GFP^+^) were degraded in the control cells but the majority of puncta in TFEB knockdown cells still were yellow, indicating a defect of lysosomal delivery (which would result in quenching of the GFP signal) in control cells. Addition of lysosomal protease inhibitors E64d and pepstatin A (E/P), which hinder lysosome-dependent autophagic degradation, led to blocked degradation of GFP for more yellow puncta accumulation in both control and knockdown cells. These results suggested that inhibition of TFEB could strikingly block flux integrity, resulting in increased yellow puncta. Furthermore, we further measured the expression of LC3 and p62, and we first found that after TFEB knockdown, the overall expression level of P62 was higher than that of control cells (Fig. 3D). More importantly, under rapamycin induction, comparable LC3-Ⅱ/LC3-Ⅰ ratio levels were detected between control and knockdown groups through inhibition of autophagosome-lysosome fusion by Bafilomycin A1 (Fig. 3D), indicating that TFEB might not have significant effect on autophagosome synthesis. Therefore, the results demonstrated that UUO-induced autophagosome degradation might be insufficient, which may be due to the TFEB deficiency mediated impaired autophagy flux integrity but not autophagosome synthesis.

Next, we characterized the effect of TFEB-mediated autophagy specifically on cell cycle progression first by using immunofluorescence staining. As depicted in Fig. 3E, following TGF-β1 treatment, there were increased p-H3-positive cells with more LC3 accumulation in the control HK-2 cells, however, the response was attenuated when TFEB was overexpressed. Subsequently, the Western blot analysis indicated that regardless of TGF-β1 alone or TGF-β1 combined with rapamycin treatment, the levels of p21, p-CDK1 and cyclin B1 in TFEB knockdown HK-2 cells were significantly higher than those in control HK-2 cells (Fig. 3F). Furthermore, the addition of E/P enhanced the expression of G2/M phase arrest proteins, while rapamycin inhibited the gene expressions in control cells, but to a lesser extent these responses in knockdown groups. More importantly, the expression levels of the proteins were comparable between control and TFEB knockdown cells after incubation with E/P (Fig. 3F). Taken together, our results indicated that the depression of TFEB impeded renal autophagy flux integrity, which led to G2/M cell cycle arrest.

### TFEB enhances the expression of ATP6V0C via directly binding to the ATP6V0C promoter

**Figure 4:**
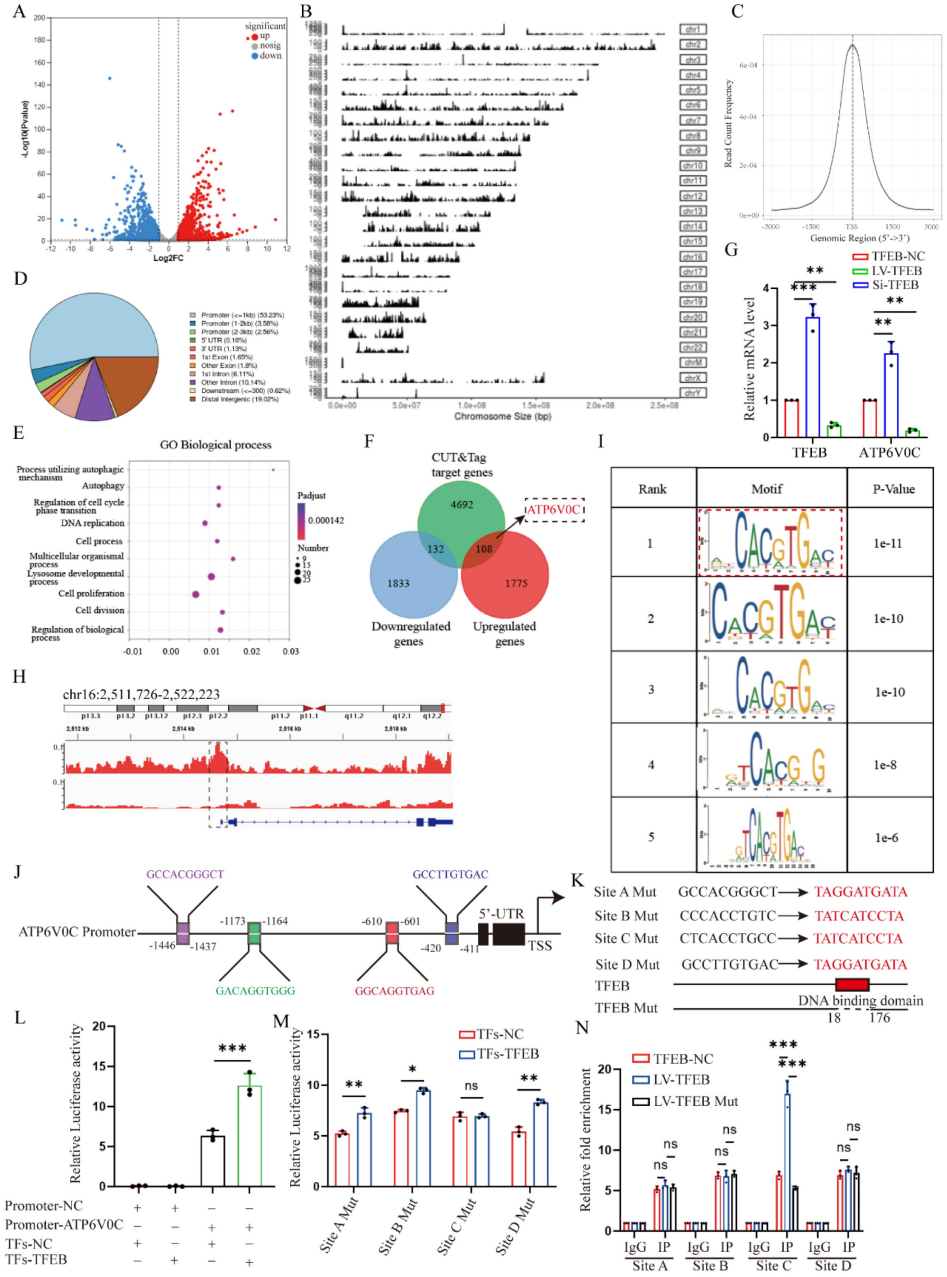
** TFEB directly induces the ATP6V0C transcription by binding to the ATP6V0C promoter at Site C.** (A) Volcano Plot analysis of the DEGs from RNA-seq between TFEB-NC and overexpression in HK-2 cells pretreated with TGF-β1. (B) A summary of the distributions of the ChIP peaks over different chromosomes by CUT&Tag technology. (C) The distribution of the reads around the transcription start sites (TSS) of different genes. (D) Pie diagram and the ratios of the TFEB-binding peaks located at different regions. (E) Bubble plotted for the peak-related target DEGs enriched in the different biological processes by GO analysis. (F) The Venn diagram of overlapping genes between DEG sets through RNA-seq and the target gene set identified by CUT&Tag. (G) Validation of the regulation of ATP6V0C expression through TFEB using RT-qPCR. (H) TFEB peak enrichment in the promoter region of the ATP6V0C gene by CUT&Tag analysis. (I) Five predicted TFEB-binding motifs with the most significant differences among peaks. (J) The predicted four binding sites in the ATP6V0C promoter from the JASPAR database. (K) The sequences of four binding sites and the scheme of the mutations. (L) Validation of the binding of TFEB to the ATP6V0C promoter by using dual-luciferase assay. (M) Dual-luciferase assay of the TFEB binding changes for the mutation of four sites in the ATP6V0C promoter. (N) Validation of the binding of TFEB expression or mutation to the four sites in ATP6V0C promoter through CUT&Run-qPCR. Data are means ± SD at 3 independent experiments; ns, not significant, *p < 0.05, **p < 0.01, ***p < 0.001.

Considering that TFEB functions greatly in regulating various cellular processes as a major transcription factor, we further explored the potential downstream targets via further combining RNA-seq and ChIP-seq. First, the RNA-seq analysis indicated that as many as 3848 genes were differentially expressed between TFEB control and overexpression groups (Fig. 2A), among which 1883 genes were upregulated and 1965 were downregulated (|fold change| ≥ 1.5, P < 0.05) (Fig. 4A). Then, we studied the genome-wide target sites of TFEB using the CUT&Tag analysis approach and found 9610 peaks corresponding to 4932 RefSeq genes, and the distribution of these peaks is shown in Fig. 4B and 4C. Most TFEB peaks were preferentially located close to transcriptional start sites (TSSs) of genes, with more than 55% of these peaks being located in promoter regions (Fig. 4D). The GO analysis of the peak-related genes indicated that the TFEB target DEGs were involved in various biological processes, including DNA replication, regulation of cell cycle and autophagy, which was consistent with the findings that TFEB promoted the autophagy flux and blocked G2/M arrest of cells by RNA-seq analysis (Fig. 4E and 2B). Next, we together RNA-seq with CUT&Tag analysis to investigate the overlapping gene sets among the DEGs after TFEB overexpression. We found that 108 upregulated genes and 132 downregulated genes were included in the set of TFEB target genes (Fig. 4F). Normal autophagy flux depends on functional lysosomes. Interestingly, the expression of ATP6V0C, a key molecule associated with lysosomal biogenesis, was positively and proportionally correlated with the expression of TFEB (Fig. 4F and 4G). More importantly, TFEB peaks well enriched in the ATP6V0C promoter region (Fig. 4H).

Next, we detected whether TFEB could directly up-regulate ATP6V0C expression in HK-2 cells. The motifs in common between the peaks were detected, and Fig. 4I shows the top five motifs with the most significant differences. According to the predicated motifs and JASPAR database, we selected the first matrix profile MA0692.1 as potential binding site for further analysis and predicted four positive strand sequences based on their scores as binding sites for TFEB in the ATP6V0C promoter (Fig. 4I and 4J). The four putative wild and mutant binding sites between TFEB and ATP6V0C promoter are illustrated in Fig. 4K. To corroborate these findings, dual-luciferase reporter gene assays first showed that co-transfection with TFEB expression and the ATP6V0C promoter-controlled luciferase expression plasmids in HEK293T cells remarkedly enhanced the ATP6V0C promoter-controlled luciferase expression (Fig. 4L). Further Dual-luciferase reporter that co-transfection with the TFEB expression and a ATP6V0C promoter site-specific mutant plasmids in HEK293T cells verified that the mutant sites of A, B, and D abrogated the TFEB-enhanced reporter luciferase activity, whereas this change was not developed after mutating the site C at ATP6V0C promoter (Fig. 4M). Additionally, CUT&Run-qPCR was performed, and the results similarly confirmed that compared with the negative control IgG, anti-TFEB antibody significantly precipitated the ATP6V0C promoter region containing the C site but not the other three sites in HK-2 cells (Fig. 4N). Collectively, these results showed that TFEB directly bound to the ATP6V0C promoter at Site C to enhance the expression of ATP6V0C.

### ATP6V0C functions as a characterized lysosome biogenesis to promote autophagic flux and decrease G2/M cell cycle arrest

**Figure 5:**
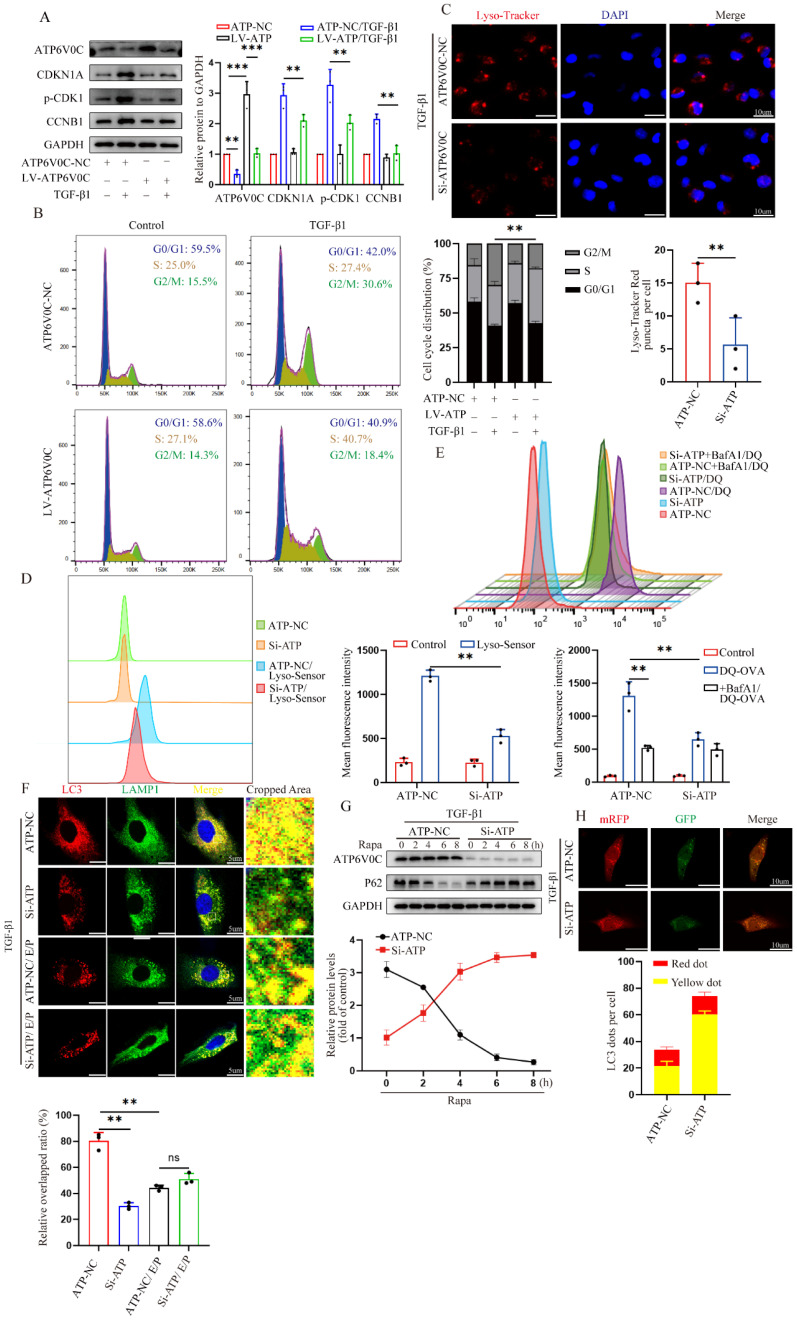
** ATP6V0C functions as a characterized lysosome biogenesis to promote autophagic flux and decrease G2/M cell cycle arrest.** (A) Immunoblot and quantitative analysis of ATP6V0C, CDKN1A, p-CDK1 and CCNB1 in ATP6V0C-NC or overexpression HK-2 cells incubated with or without TGF-β1. (B) Cell cycle distribution by flow cytometry analysis in ATP6V0C-NC or overexpression HK-2 cells incubated with or without TGF-β1. (C) Lyso-Tracker Red staining in ATP6V0C-NC or knockdown HK-2 cells treated with TGF-β1, and quantification of Lyso-Tracker Red punctas. Scale bar, 10 μm. (D) Lyso-Sensor Green staining or not staining in ATP6V0C-NC or knockdown HK-2 cells upon TGF-β1, and the Half Offset Histogram and quantification of fluorescence intensity by flow cytometry. (E) FITC-labeled DQ-OVA fluorescence intensity measured by flow cytometry in HK-2 cells upon TGF-β1 treated with or without BafA1, and Stagger Offset Histogram and quantitative analysis of mean values. (F) The colocalization for LAMP1 and LC3 in ATP6V0C-NC or knockdown HK-2 cells upon TGF-β1 treated with or without E/P, and quantitative measurement of the colocalization ratio. Scale bar, 10 μm. (G) ATP6V0C-NC or knockdown HK-2 cells were incubated with rapamycin for 0, 2, 4, 6, 8h. Immunoblot and quantitative analysis of tendency of P62. (H) The ATP6V0C-NC or knockdown HK-2 cells were transfected with mRFP‑GFP‑LC3, and quantitative analysis of red and yellow dots numbers. Scale bar, 10 μm. Data are means ± SD at 3 independent experiments; ns, not significant, *p < 0.05, **p < 0.01, ***p < 0.001.

Although ATP6V0C could be directly regulated by TFEB, little is known about its role in cell cycle regulation. To this end, we first overexpressed ATP6V0C with lentivirus plasmid in HK-2 cells. Notably, the Western blot analysis revealed that a significant decrease in the levels of p21, p-CDK1 and cyclin B1 in the ATP6V0C overexpression group after treatment with TGF-β1, as compared with their control cells (Fig. 5A), which is consistent with that in the TFEB data. Likewise, flow cytometry quantitative analysis confirmed a more than 10% decrease of the cell population in the G2/M phase in ATP6V0C overexpression cells upon TGF-β1 (Fig. 5B). Then, we elucidated its underlying mechanisms. Due to the fact that the completion of autophagic flux depends on normal lysosome function and autophagosome-lysosome fusion, and ATP6V0C is a subunit in the V-ATPase multi-subunit complex, which mainly plays a major role in achieving the activity of lysosomal hydrolase to maintain acidic function, we first tested if ATP6V0C is required for acidification of lysosomes in HK-2 cells upon TGF-β1. Lyso-Tracker Red and Lyso-Sensor Green were applied to assess lysosomal pH. As shown in Fig. 5C, knockdown ATP6V0C with ATP6V0C-siRNA resulted in a notable decrease in the quantity of red puncta observed in Lyso-Tracker staining. Further, flow cytometry was performed to qualitatively detect the average green fluorescence intensity in Lyso-Sensor staining, and the results similarly showed that green fluorescence intensity in ATP6V0C knockdown group was significantly decreased (Fig. 5D). To further comprehensively apprehend the effect of ATP6V0C on lysosomal acid degradation fate, we measured the ability of ATP6V0C knockdown cells to degrade DQ-OVA, a visualizable self-quenched marker 34-36. The result suggested that the acid degradation-induced DQ-OVA fluorescent signal was reduced in ATP6V0C knockdown HK-2 cells, and the addition of the lysosomal acidification inhibitor bafilomycin A1 in control cells yielded a comparable outcome in terms of inhibiting DQ-OVA fluorescence (Fig. 5E). Therefore, these data indicate that ATP6V0C have notable roles in activation of V-ATPase-driven lysosome acidification and degradation.

Subsequently, we proceeded with the assessment of process in the fusion of autophagosomes and lysosomes. To test this, autophagosomes were labeled with anti-LC3 and lysosomes were labeled with anti-LAMP1, and the results revealed that the co-localization of autophagosomes and lysosomes in ATP6V0C knockdown group was significantly reduced than that in control group, whereas the difference was abolished by the addition of E/P (Fig. 5F). Furthermore, the Western blot result showed that p62 was gradually degraded in a time-depend manner by pretreatment with Rapa in the control group but not in the ATP6V0C knockdown group, indicating that ATP6V0C was required for autophagosome degradation (Fig. 5G).

Additionally, we also transfected mRFP-GFP-LC3 plasmid into cells. As expected, the green puncta and red puncta could be observed in both control and ATP6V0C knockdown group, but the number of yellow puncta was remarkedly increased in ATP6V0C knockdown cell compared with control cells (Fig. 5H), suggesting the disruption of autophagosomes and lysosomes interaction. Collectively, these data suggest that ATP6V0C could exert prominent lysosome acidification and degradation function and promote autophagosome-lysosome fusion.

### TFEB induces autophagic flux integrity, mainly dependent on scaffold protein ATP6V0C-mediated autophagosome-lysosome fusion

**Figure 6:**
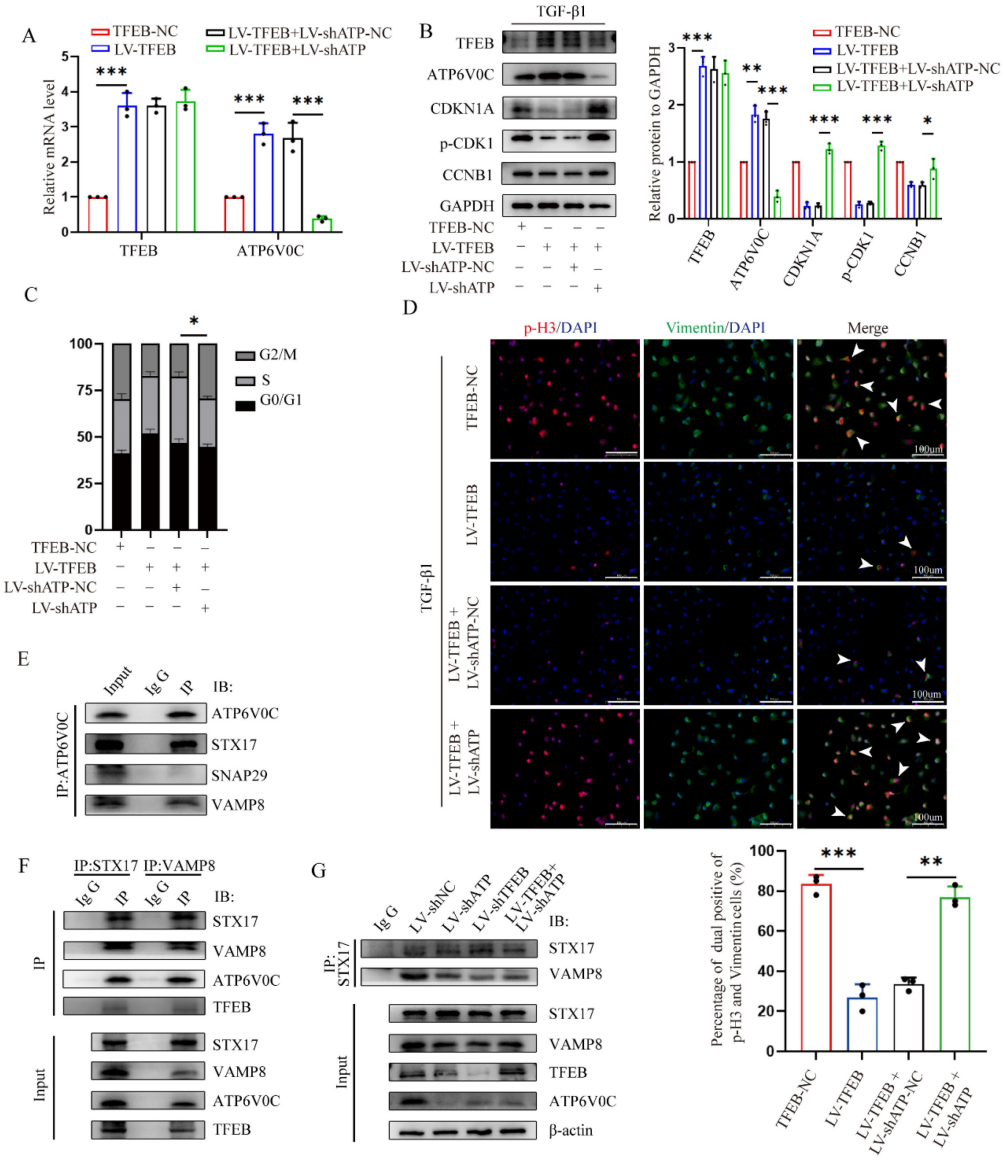
** TFEB mainly depends on scaffold protein ATP6V0C-mediated autophagosome-lysosome fusion to promote the autophagic flux integrity.** (A-D) ATP6V0C-specific shRNA (LV-shATP6V0C) or control empty vector shRNA (LV-shATP6V0C-NC) were transfected into TFEB-NC or overexpressed HK-2 cells. (A) The mRNA levels of TFEB and ATP6V0C expression were confirmed by RT-qPCR. (B) Immunoblot and quantitative analysis of TFEB, ATP6V0C, CDKN1A, p-CDK1 and CCNB1, (C) flow cytometry analysis of the cell cycle distribution, and (D) double immunofluorescence staining and quantitative analysis of dual positive of p-H3 and Vimentin in the cells treated with TGF-β1. Scale bar, 100 μm. The arrows indicate dual positive cells. (E) Western blot of whole-cell lysates (WCLs) and Co-IP was performed using antibodies against ATP6V0C, STX17, SNAP29 or VAMP8, and captured samples were immunoblotted with antibodies against ATP6V0C. IgG served as the negative control. (F) Immunoblot of WCLs and Co-IP was performed using antibodies against STX17, VAMP8, ATP6V0C or TFEB, and captured samples were immunoblotted with anti-STX17 and anti-VAMP8. IgG served as the negative control. (G) Control shRNA, LV-shATP6V0C or LV-shTFEB were transfected into HK-2 cells, and LV-shATP6V0C was transfected into TFEB-overexpressed HK-2 cells. Western blot of WCLs using antibodies against STX17, VAMP8, ATP6V0C or TFEB, and the WCLs were immunoprecipitated with anti-STX17, subjected to immunoblot and probed with either anti-STX17 or anti-VAMP8. IgG served as the negative control. Data are means ± SD at 3 independent experiments; ns, not significant, *p < 0.05, **p < 0.01, ***p < 0.001.

To further evaluated investigate whether TFEB inhibited the ECM deposition through ATP6V0C up-regulation, we first tested whether ATP6V0C silencing could mitigate or reverse the reduced G2/M cell cycle arrest by TFEB overexpression in the presence of TGF-β1. We stably knocked down ATP6V0C by lentivirus transduction with ATP6V0C-specific shRNAs in TFEB over-expressed HK-2 cells confirmed by RT-PCR and western blot (Fig. 6A and 6B). Functionally, we found that ATP6V0C knockdown restored the levels of p21, p-CDK1 and cyclin B1 expression in the TFEB overexpressed HK-2 cells upon TGF-β1 (Fig. 6B). Similarly, ATP6V0C knockdown also abrogated the reduced percentage of cells in G2/M phase in TFEB overexpressed cells (Fig. 6C). More importantly, TFEB overexpression decreased p-H3-positive cells and Vimentin accumulation, which were reversed by ATP6V0C knockdown (Fig. 6D). These results suggest that TFEB could inhibited the G2/M cell cycle arrest and ECM deposition in a ATP6V0C-dependent manner.

Next, we further sought to characterize the specific mechanism by which TFEB depends on ATP6V0C. As expected, TFEB overexpression resulted in a significant elevation in the number of red puncta compared to the control cells in Lyso-Tracker staining (Fig. S1A), suggesting that TFEB also functions to enhance the acidification of lysosomes. Intriguingly, we found that ATP6V0C silencing did not apparently abrogated the enhanced red puncta in the TFEB over-expressed cells, and the Lyso-Sensor staining confirmed the finding (Fig. S1A and S1B). The results indicated that ATP6V0C-mediated lysosome acidification might not be directly correlated with the TFEB-mediated lysosome acidification, implying that ATP6V0C-mediated lysosome acidification did not play a crucial role of in TFEB-induced ECM processing. Moreover, TFEB overexpression induced DQ-OVA fluorescent signal, which was also not inhibited by ATP6V0C knockdown (Fig. S1C). Thus, these data implied that TFEB-induced autophagic flux integrity may be independent of ATP6V0C-mediated lysosome acidification and degradation.

As the lysosome function was intact in the absence of ATP6V0C, we subsequently investigated whether TFEB could promote autophagosome-lysosome fusion progression through ATP6V0C. And we first asked if ATP6V0C could interact with SNAREs, a very important complex directly involved in the final autophagosome-lysosome membrane fusion 37. Mechanistically, we immunoprecipitated cell lysates with anti-ATP6V0C and found that ATP6V0C could interacted with STX17 and VAMP8 but not SNAP29 (Fig. 6E). Similarly, immunoprecipitants with anti-STX17 or anti-VAMP8 followed by Western blot analysis with anti-ATP6V0C confirmed this interaction (Fig. 6F). Specially, when subjected to immunoblotting with anti-TFEB, the results demonstrated that there was indeed no physical interaction between TFEB and SNAREs (Fig. 6F). Thus, these results indicated that ATP6V0C, but not TFEB, could associated with SNARE complex well. Additionally, STX17 and VAMP8 are important members of the SNARE, and their binding extent can illustrate the strength of autophagosome-lysosome fusion 38, 39. We found that both ATP6V0C and TFEB silencing attenuated the association of STX17 with VAMP8, whereas the weakened interaction caused by ATP6V0C was not restored through the overexpression of TFEB (Fig. 6G), indicating that TFEB promoted the linkage mainly through ATP6V0C. Collectively, our results suggested that ATP6V0C, might acting as a scaffold protein in bridging STX17 and VAMP8, which was critical to TFEB-mediated autophagosome-lysosome fusion.

### DNMT3a-mediated TFEB promoter is hypermethylated, and demethylated by 5A-za recover fibrotic TFEB loss and alleviate renal fibrosis pathogenesis

**Figure 7:**
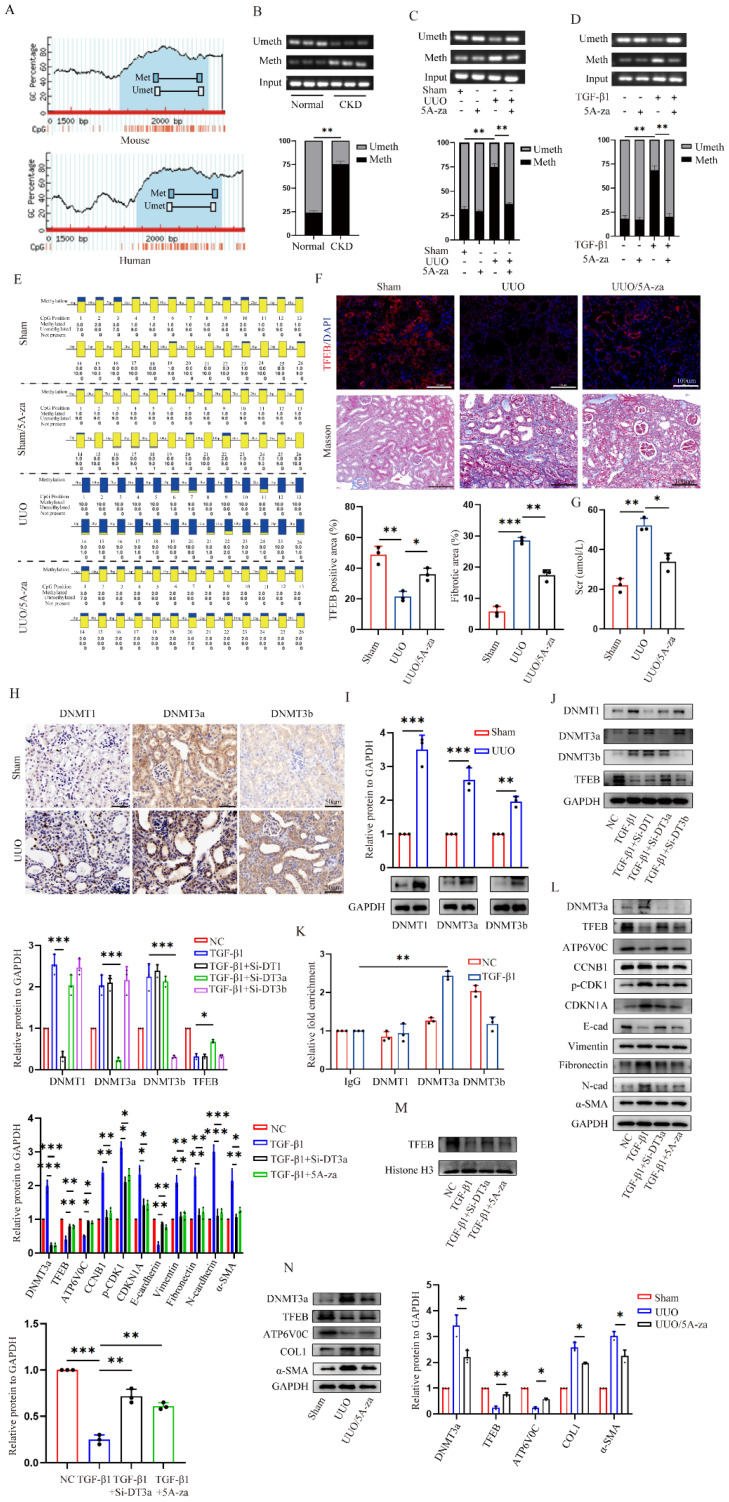
** 5-Aza recovers DNMT3a-mediated TFEB promoter hypermethylation, and alleviates renal fibrosis pathogenesis.** (A) Schematic diagram of mouse and human TFEB promoters. Guanine and cytosine (GC) contents are indicated. (B-D) The methylation profile of the TFEB gene from the MSP results. The agarose gel electrophoresis and quantitative analysis of MSP products from (B) renal tissues of CKD patients and normal controls, (C) renal tissues of sham and UUO mice treated with or without 5A-za, and (D) HK-2 cells treated with or without TGF-β1 and 5A-za. (E) The TFEB methylation profile from the BSP analysis of mouse renal tissues as in (C). (F-G) Representative immunofluorescence staining of TFEB (F), Masson trichrome staining (F) and serum creatinine level (G) from renal tissues of sham and UUO mice treated with or without 5A-za, and quantifications of TFEB and fibrotic positive areas. Scale bar, 100 μm. (H-I) Representative IHC and immunoblot of TFEB content in sham and UUO mouse renal tissues. Scale bar, 200 μm and 50 μm. (J) TGF-β1-treated HK-2 cells were transfected with DNMT1, DNMT3a, or DNMT3b siRNA. The protein levels of TFEB and DNMTs were analyzed by immunoblot. (K) The interaction between DNMT1, DNMT3a, DNMT3b and TFEB promoter through CUT&Run-qPCR. (L-M) TGF-β1-treated HK-2 cells were transfected with Si-DNMT3a or incubated with 5A-za. Immunoblot and quantitative analysis of DNMT3a, TFEB, ATP6V0C, CDKN1A, p-CDK1, CCNB1 and EMT-related protein levels in whole cell lysates and TFEB protein levels in nuclear extractions (M). (N) Immunoblot and quantitative analysis of DNMT3a, TFEB, ATP6V0C, COL1 and α-SMA from renal tissues of sham and UUO mice treated with or without 5A-za. Data are means ± SD, each experiment was performed in triplicate; ns, not significant, *p < 0.05, **p < 0.01, ***p < 0.001.

Finally, we evaluated the underlying mechanism behind the low expression of TEFB in UUO-induced fibrotic kidneys. We first analyzed TFEB promoter by MethPrimer software and found both mouse and human TFEB promoters are enriched with typical CpG islands (Fig. 7A). Thus, we decided to test the promoter DNA methylation status by methylation specific PCR (MSP), and surprisingly found that the methylation levels at CpG island of TFEB in the kidneys of UUO mice and CKD patients were significantly higher than those in the normal groups (Fig. 7B and 7C). To confirm that, the UUO mice treated with 5A-za, a strong DNA demethylating agent, exhibited a significant inhibition of methylation level at the CpG island of TFEB (Fig. 7C). Additionally, similar *vitro* experiments also confirmed this finding (Fig. 7D). Furthermore, we also analyzed the mouse kidneys by bisulfite-sequencing PCR (BSP), the gold standard for DNA methylation assessment. Consistently, the results demonstrated that a substantial increase in the CpG island methylation level of TFEB in UUO mice, whereas 5A-za treatment lowered the level (Fig. 7E). More importantly, the treatment by 5A-za noticeably recovered TFEB level (Fig. 7F), and effectively attenuated the elevated collagen and serum creatinine level, comparing to UUO mice (Fig. 7F and 7G). These results indicate that 5A-za could preserve TFEB hypermethylation and loss and alleviate kidney fibrotic pathologies.

To further identify the upstream events leading to the TFEB promoter hypermethylation, we detected all three bioactive DNA methyltransferases (DNMTs) and found that DNMT1, DNMT3a and DNMT3b levels were all significantly increased in kidneys of UUO mice (Fig. 7H). The Western blot analysis also confirmed the similar results (Fig. 7I). To further analyze which DNMTs influence the methylation of TFEB, DNMT1, DNMT3a, and DNMT3b were knockdown with siRNA in HK-2 cells. The Western blot analysis showed that DNMT3a knockdown impressively reversed the low expression of TFEB induced by TGF-β1, while other DNMT1 and DNMT3b did not (Fig. 7J). Further, CUT&Run-qPCR was performed and the results showed that only DNMT3a could successfully pull down the TFEB gene during ECM process, while DNMT1 and DNMT3b could not (Fig. 7K). Next, we silenced DNMT3a with Si-RNA and 5A-za to verify the changes of TFEB, and the results showed that total TFEB protein expression in whole cell increased significantly after DNMT3a silencing (Fig. 7L), moreover, the levels of ATP6V0C, p21, p-CDK1, cyclin B1 and ECM-related protein also had a significant amelioration (Fig. 7L). We also found that TGF-β1-inhibited TFEB nuclear translocation was obviously restored by 5A-za and Si-DNMT3a treatment (Fig. 7M), indicating the recovery of TFEB dephosphorylation and activation. In addition, the *vivo* DNMT3a inhibition by 5A-za also confirmed the repression of DNMT3a recovered TFEB loss and prevent mouse kidney fibrogenesis (Fig. 7N). Taken together, these results confirmed that DNMT3a-mediated TFEB promoter was hypermethylated, and demethylated by 5-Aza could recover fibrotic TFEB loss and alleviate renal fibrosis pathogenesis.

## Discussion

As a signal initiated to maintain cellular homeostasis, autophagy has garnered significant attention in the field of cell stress or nutrient deprivation 15. TFEB stimulates a wide range of autophagy- and lysosome-related genes and has been shown as a vital host response against various cellular stress 21. Nevertheless, the regulatory mechanism of biological function and expression level of TFEB in UUO-induced renal fibrogenesis remained elusive. In the present study, we identify steps in autophagy flux in the TFEB protective pathway compromised by UUO-induced renal fibrosis. We found that the UUO-induced renal fibrosis decreased TFEB proteins and its downstream ATP6V0C-mediated lysosomal biogenesis, resulting in insufficient autophagy and further tubular cell G2/M arrest and ECM deposition, and revealed that TFEB effectively restored the disruption of autophagosome-lysosomal fusion and disorder in autophagy flux in an ATP6V0C-dependent manner through bridging with SNAREs. In addition, we also demonstrated that the overexpression of TFEB in kidney or TFEB promoter demethylation with 5A-za could effectively alleviate renal fibrosis pathogenesis.

CKD is a renal dysfunction process of renal progressive injury, and many cellular and molecular events, including inflammation infiltration, EMT and/or endothelial-mesenchymal transition and ECM deposition, occur during CKD 40. It is worth noting that autophagy can also significantly contribute to renal fibrosis following injury 41. TFEB is a crucial regulator of autophagy signaling in various mammalian cells. In a recent study aimed at examining the role of TFEB in renal fibrosis reported that TFEB expression increased in renal tubules in adenine-induced renal injury mice 42.

This may be the fact that the mechanism of adenine-induced CKD is through extended oxidative stress and pro-inflammatory factors 43 and TFEB is a protective transcription factor against cell inflammation 44 and TFEB promoted autophagy process and improved kidney injury by reducing inflammation 45. Consistent with these previous findings, we used the UUO model, which exhibited that TFEB was initially increased in renal tubular cells of obstructed kidneys at the early inflammatory stress stage, which further extend the hypothesis that TFEB is a potential anti-fibrosis factor.

Recently, the growing evidence has suggested that TFEB has a crucial role in various chronic disease and fibrosis. For instance, it has been found that administration of Torin-1 decreased chronic liver steatosis and injury through elevating TFEB level. Moreover, overexpression of TFEB in the liver of mice was found to mitigate the severity of ethanol-induced liver injury 24. In addition, recent studies also showed that impaired TFEB promoted the development of chronic pancreatitis and pulmonary fibrosis in mouse and human 22, 23. To more profoundly determine the effect of TFEB in renal fibrosis, we surprisingly found the TFEB declined to less basal levels at d 14 after UUO. These expression differences indicated that the actual function of TFEB may depend on the specific type and different stage of fibrotic disease 46, 47. More importantly, decreased TFEB has also been observed in UUO-induced CKD patients. Thus, we also investigated whether TFEB overexpression could alleviate UUO-induced renal fibrosis. In the current reports, we confirm and extend these inferences by indicating that overexpression of TFEB in UUO-induced mice dramatically alleviated renal ECM deposition. Moreover, we noticed silencing of TFEB aggravated the TGF-β1-induced EMT transition *in vitro*, while overexpression TFEB significantly alleviated the trend of EMT transition. Indeed, the regulation of TFEB is finely tuned, during starvation, MTORC1 is released from the lysosomal membrane and TFEB is activated, dephosphorylated and translocated to the nucleus, where it promotes target gene transcription 27, 48, 49. Therefore, we not only investigated the role of TFEB in renal fibrosis from the overall expression level of TFEB, but also indicated the level of nuclear alterations in TFEB. Taken together, our findings supported the view that inhibitory effects of TFEB on UUO-induced renal fibrosis.

TFEB, a member of the MITF family along with TFE3, has been recognized as a vital transcriptional regulator of autophagy-lysosome gene expression, and this is attributed to the presence of CLEAR sequences in many genes crucial for lysosome biogenesis and function 50, 51. Moreover, TFEB and TFE3 exhibit overlapping functions and are implicated in genetic rearrangements that regulate DNA damage, proliferation and drive the formation of tumor cell 52-54. Surprisingly, our research found that TFEB was involved in regulation of the cell cycle through RNA-seq analysis. Remarkably, previous studies have demonstrated that TFEB knockdown leads to reduced phosphorylation levels of CDK4 and RB1 in endothelial cells, ultimately resulting in G1/S arrest 55. However, the difference is that we found that TFEB can block G2/M arrest of tubular cells, and the percentage of tubular cells arrested at G2/M is elevated in fibrotic kidneys, which is consistent with a previous study that suggested that rescuing the inhibition of the cell G2/M arrest can prevent renal fibrosis 56. Additionally, some data also revealed that TFEB knockdown-mediated regulation of numerous genes were targeted by canonical cell cycle regulatory transcription factors, including MYC, E2F, and FOXM1 57-59. However, it is notable that the mechanism behind cell cycle regulation by TFEB has not yet been elucidated. Interestingly, the recent research found that TFEB governs the expression of genes associated with DNA damage repair, apoptosis, and cell cycle in breast cancer cells, while lysosomal-autophagic function was independent of the prosurvival activity of TFEB in the cell 53. Instead, we found that administration with the E/P, a block the lysosome-dependent autophagic degradation, enhanced the expression of G2/M phase arrest proteins, while the autophagy activator rapamycin inhibited the gene expression* in vitro*. Thus, our findings first supported the view that autophagy genes contribute to the precise cell cycle progression mechanisms 60. Notably, when TFEB is knocked out, these responses became silenced, suggesting that TFEB-mediated autophagy plays an important role in cell cycle phase. As a result, our findings serve as the initial evidence of an interplay between TFEB, autophagy, and cell cycle regulation. Moreover, we demonstrate that TFEB-mediated blocking of G2/M arrest relies on the presence of autophagy.

The previous studies have shown that TFEB is not only involved in autophagy but also lysosomal biogenesis and exocytosis 51. TFEB can identify similar target sequences (30-CANNTG-50) and bind DNA as homodimers or heterodimers with other family members 61 and activate downstream genes and promote their transcription. To explored the potential downstream targets, we found that TFEB peaks well enriched in the ATP6V0C promoter region by using ChIP-seq. This is consistent with the finding that the expression of genes encoding the subunits of V-ATPase, which are functionally related, is modulated by MITF 62. Functionally, the primary role of V-ATP is to acidify the lysosomes of eukaryotic intracellular organelles, which is crucial for normal cellular processes such as protein degradation, antigen presentation, and intracellular membrane trafficking 63, so we investigated whether ATP6V0C activation could promote lysosomal acidification and degradation functions. Likewise, we not only observed that ATP6V0C could drive lysosome acidification and degradation, but also further found that it significantly promoted autophagosome-lysosome fusion, which was consistent with previous findings that V-ATPase may cooperate or direct interaction with SNARE proteins at the late stage of membrane fusion 64, 65. In mammalian studies, SNAREs mainly refer to syntaxin17 (STX17), synaptosomal-associated protein 29 (SNAP29), and vesicle-associated membrane protein 8 (VAMP8) 66. Interestingly, not only did we find that ATP6V0C can interacts with STX17 and VAMP8, where ATP6V0C may function as a bridging protein, we also found that ATP0C can promote the binding of the both, while TFEB cannot, which indicated that TFEB-mediated autophagic flux integrity mainly relied on ATP6V0C to well strengthen the membrane fusion of autophagosome and lysosome.

DNA methylation, as an epigenetic regulation, is a molecular link between environmental factors and complex diseases, including renal fibrosis and cancer 67-69. Importantly, recent studies suggested that DNA methylation can lead to TFEB expression silencing 70, 71, thus suggesting that it may play a crucial role in determining autophagy and renal fibrosis. To evaluate the underlying mechanism behind the low expression of TEFB in UUO-induced fibrotic kidneys, supporting this hypothesis, our data clearly showed that although DNMTs (including DNMT1, DNMT2, and DNMT3) expressions were elevated in fibrotic kidneys, and only DNMT3a could successfully pull down the TFEB gene through ChIP assay, indicating that DNMT3a is an important regulator in TFEB methylation during ECM process. More importantly, our results show that the TFEB recovery by 5-Aza, a strong DNA demethylating agent, reduced renal fibrotic pathologies by normalizing autophagy, cell cycle regulation and ECM deposition both *in vitro* and *in vivo*.

In conclusion, our study emphasized for the first time that impaired TFEB-mediated autophagosome-lysosome fusion disorder, tubular cell G2/M arrest and renal fibrosis appear to be sequentially linked, and modulation of DNA methylation-mediated TFEB expression may potentially serve as a promising therapeutic approach to prevent the advancement of UUO-induced renal fibrosis.

## Supplementary Material

Supplementary figure.

## Figures and Tables

**Figure 8:**
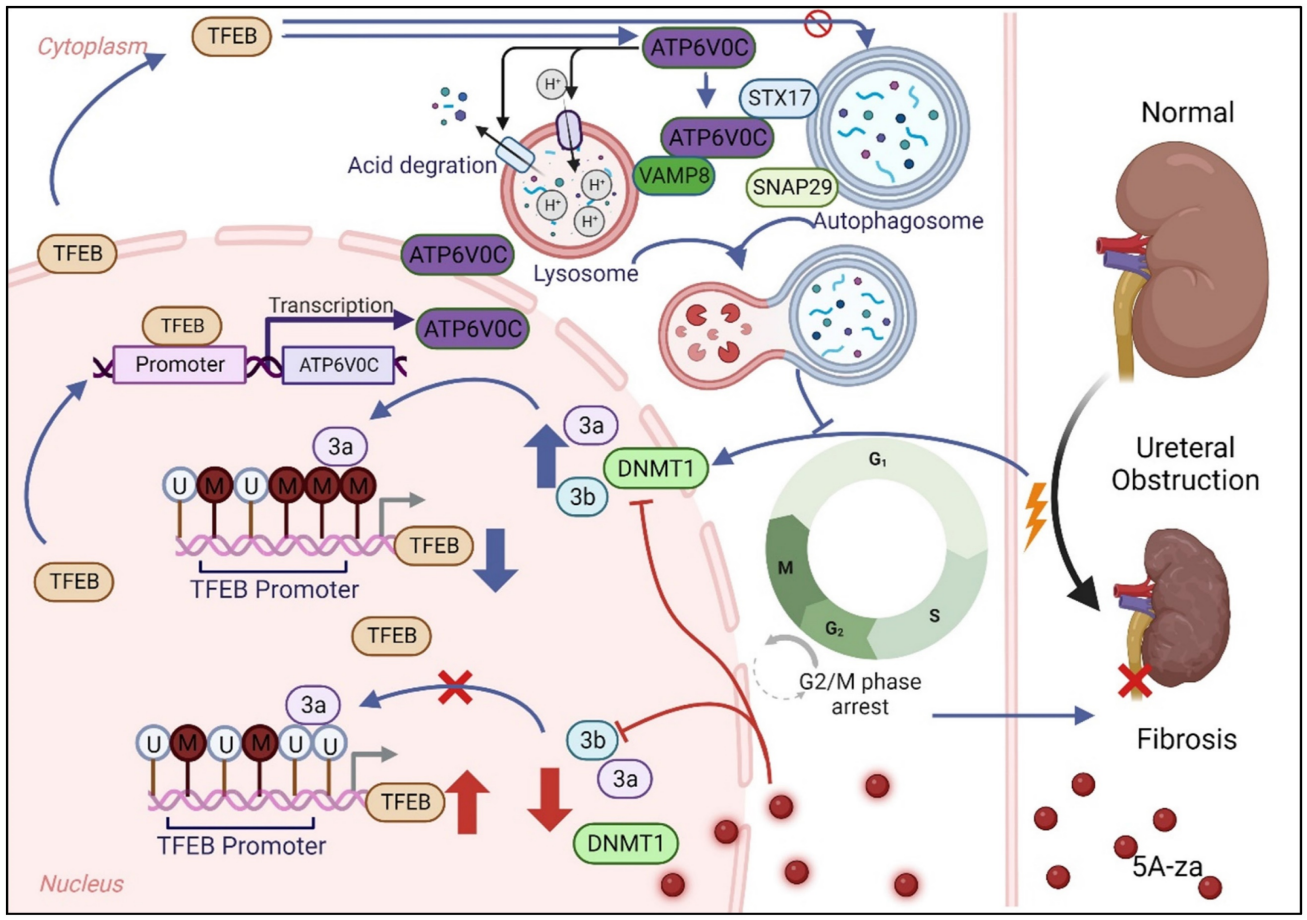
Schematic diagram showing major findings in this study.
