# Supplementary material for: Impaired TFEB-mediated autophagy-lysosome fusion promotes tubular cell cycle G2/M arrest and renal fibrosis by suppressing ATP6V0C expression and interacting with SNAREs
Source: Int J Biol Sci. 2024 Mar 3;20(5):1905–26. doi: 10.7150/ijbs.91480 (PMC10929200; doi:10.7150/ijbs.91480)
Supplement: Supplementary file 1 — Supplementary figure. [file ijbsv20p1905s1.pdf]

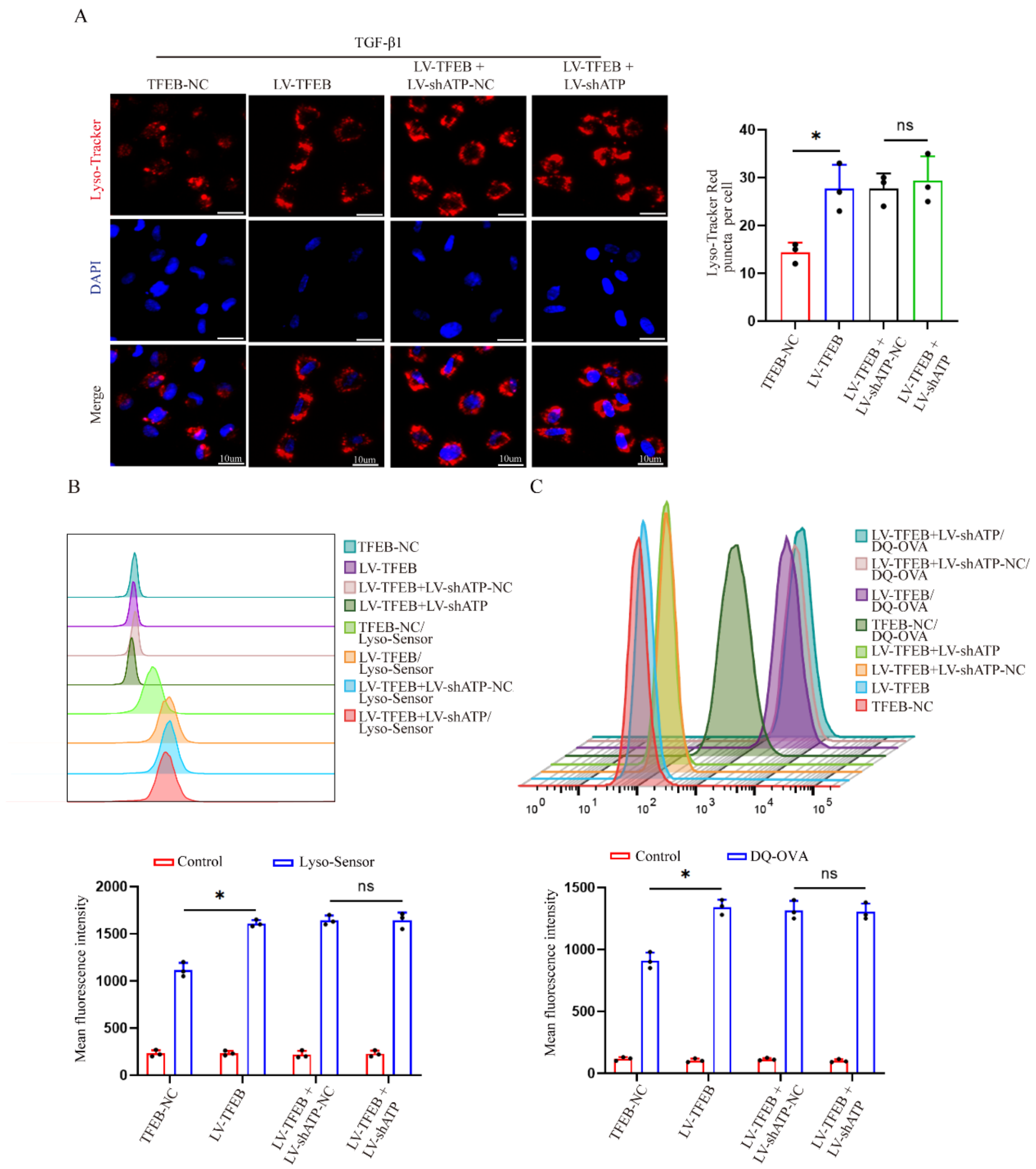


**Suplemental Figure1. TFEB-induced lysosome function may not be dependent of ATP6V0C-mediated lysosome acidification and degradation.** (A) ATP6V0C-specific shRNA (LV-shATP6V0C) or control empty vector shRNA were transfected into TFEB-control or overexpressed HK-2 cells and then were treated with TGF-β1. Lyso-Tracker Red staining in the cells and quantification of Lyso-Tracker Red puncta. Scale bar, 10 μm. (B) Lyso-Sensor Green staining or not staining in the cells, and the Half Offset Histogram and quantification analysis of fluorescence intensity by flow cytometry. (C) The cells were treated with or without FITC-labeled DQ-OVA, and the mean values of fluorescence intensity were analyzed by flow cytometry in a manner of Stagger Offset Histogram.
